# Experimental Evidence of the Antitumor, Antimetastatic and Antiangiogenic Activity of Ellagic Acid

**DOI:** 10.3390/nu10111756

**Published:** 2018-11-14

**Authors:** Claudia Ceci, Pedro M. Lacal, Lucio Tentori, Maria Gabriella De Martino, Roberto Miano, Grazia Graziani

**Affiliations:** 1 Department of Systems Medicine, University of Rome Tor Vergata, 00173 Rome, Italy; claudiaceci@hotmail.it (C.C.); tentori@uniroma2.it (L.T.); 2 Laboratory of Molecular Oncology, Istituto Dermopatico dell’Immacolata, IDI-IRCCS, 00167 Rome, Italy; p.lacal@idi.it; 3 Laboratory of Medicinal Chemistry, Department of Biomedicine and Prevention, University of Rome Tor Vergata, 00173 Rome, Italy; maria.gabriella.de.martino@uniroma2.it; 4 Urology Unit, Department of Surgical Sciences, University of Rome Tor Vergata, 00173 Rome, Italy; mianor@virgilio.it

**Keywords:** polyphenolic compounds, colorectal cancer, breast cancer, prostate cancer, non-small cell lung cancer (NSCLC), melanoma, bladder cancer, ovarian cancer, metastases, angiogenesis

## Abstract

Ellagic acid (EA) is a naturally occurring polyphenolic compound endowed with strong antioxidant and anticancer properties that is present in high quantity in a variety of berries, pomegranates, and dried fruits. The antitumor activity of EA has been mostly attributed to direct antiproliferative and apoptotic effects. Moreover, EA can inhibit tumour cell migration, extra-cellular matrix invasion and angiogenesis, all processes that are crucial for tumour infiltrative behaviour and the metastatic process. In addition, EA may increase tumour sensitivity to chemotherapy and radiotherapy. The aim of this review is to summarize the in vitro and in vivo experimental evidence supporting the anticancer activity of pure EA, its metabolites, and EA-containing fruit juice or extracts in a variety of solid tumour models. The EA oral administration as supportive therapy to standard chemotherapy has been recently evaluated in small clinical studies with colorectal or prostate cancer patients. Novel formulations with improved solubility and bioavailability are expected to fully develop the therapeutic potential of EA derivatives in the near future.

## 1. Introduction

**Figure 1:**
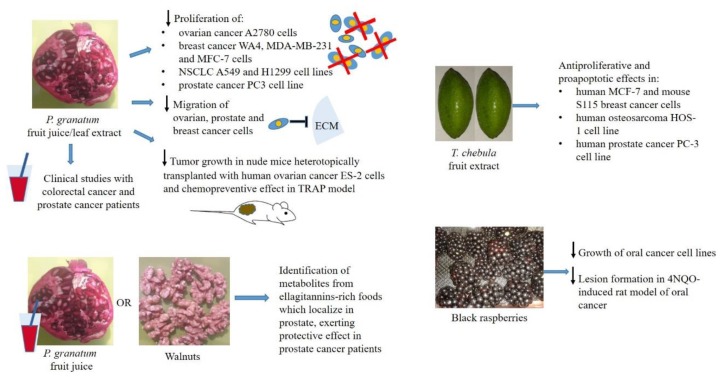
Experimental evidence of the chemopreventive and therapeutic effects of EA derived from dietary fruits. ECM, extracellular matrix; ↓, inhibition.

Ellagic acid (EA) is a polyphenolic compound from the family of ellagitannins (ETs), historically identified as the active compound responsible for the antioxidant properties of pomegranate (*Punica granatum*). EA is a dimeric derivative of gallic acid, which may occur either as a free form or as a complex within ETs in high quantity in a variety of berries (e.g., strawberries, raspberries, blackberries, cranberries, goji berries), pomegranates, grapes, walnuts and a type of edible mushroom (*Fistulina epatica*, where EA represents about 50% of total phenolic content) [1,2]. Tannins, and specifically the sub-group of hydrolysable tannins, including ETs, gallotannins and epigallocatechin-3-gallate [3] received a growing interest in the last decades, in light of their beneficial effects on human health and activity in a number of diseases including cancer. Fruits containing EA have been tested for their anticancer potential in a variety of tumour models (Figure 1).

Table 1 shows a list of the most common sources of this polyphenolic compound from the diet, i.e., from foods and beverages, where it is present as unmodified, glycosylated and acetylated EA or as ETs polymers that can be hydrolysed to release EA [4]. Only a small fraction of free EA from the diet is absorbed in the stomach, whereas ETs, which are resistant to acid hydrolysis and degradation, release free EA in the small intestine, where it can be directly absorbed due to the neutral to slightly basic pH [4]. The uptake is thought to take place via passive diffusion driven by a concentration gradient, without the involvement of specific transporters [5]. However, as assessed in a Caco-2 cells monolayer model, absorption of EA decreases in the presence of organic anion transporting polypeptide (OATP) and sodium/glucose cotransporter 1 (SGLT1) inhibitors, suggesting that EA intake occurs not only via passive diffusion but also through protein mediated transport [6]. Then, absorbed EA is converted to methyl esters, dimethyl esters or glucuronides, and these metabolites can be detected in human plasma and urine 1–5 h after ET ingestion [7,8]. Unabsorbed ETs and free EA can reach the colon, where they are metabolized by gut microbiota to yield urolithins (Uro) that, compared to free EA, have a much higher absorption rate, presumably because of increased lipophilicity [9]. In fact, Uro are present in the bloodstream in much higher concentrations. Overall, EA shows a low bioavailability (0.1–0.4 mmol/L, equivalent to 30–120 ng/mL, with respect to 0.5–18.6 mmol/L, equivalent to 0.1–4 mg/mL for Uro) that hampers its beneficial effects to human health after in vivo oral administration.

Dietary polyphenols are not consumed in sufficient quantities among population in Western and developing countries, because of inadequate fruit and vegetable intake. Actually, the WHO recommends the consumption of at least 400 g of fruit and vegetables a day, since they may reduce the risk of cardiovascular diseases and certain types of cancer [10]. According to a recent study, in Europe, the average consumption is 375 g/day, with nine out of fourteen countries consuming less than the recommended quantity. Consequently, the average intakes of EA and other phytonutrients are higher in adults who consume >400 g/day of fruit and vegetables, compared with those who do not [11]. In the UK, it has been estimated that EA intake is about 5 mg/day; however, for some individuals, it may be 100 mg/day if pomegranate, berries or walnuts are regularly eaten. In the US, the mean intake is 5–5.9 mg/day, while the consuming recommended levels in men and women are estimated to be 17.9 and 27.6 mg/day, respectively [12]. Moreover, the intake of phytonutrients, including EA, is low among adults who consume less than the daily-recommended amount of fruit and vegetables [12]. In addition, there is huge variability in microbial metabolism of EA between individuals, depending on differences in gut microbial ecology [13].

Since a large amount of literature on EA therapeutic potential has accumulated over the past decades, the aim of this review is to summarize the current experimental evidence supporting its anticancer activity in a variety of tumour models.

## 2. In Vitro and In Vivo Activity of Ellagic Acid in Different Tumour Models

### 2.1. Ellagic Acid and Colorectal Cancer

Colorectal cancer is (CRC) the third-most common cancer worldwide and the second leading cause of death from cancer [25]. In Europe, it is the third most frequently occurring malignancy in men and the second in women, whereas in the US it is the third most common cancer diagnosed in both men and women. In about 95% of cases, the histological type is adenocarcinoma [26,27].

The first studies testing EA anticancer properties against CRC were done in vitro with human colon cancer cell lines and date back to the beginning of 2000. In colon cancer SW480 cells EA induced growth inhibition as a consequence of DNA damage that resulted in p53 and p21 activation, and down-regulation of insulin like growth factor-II (IGF-II) [28]. Some years later, EA antiproliferative activity and cytotoxicity were shown to be specific for cancer cells, since it induced apoptosis in colon cancer Caco-2 cells but did not show any toxic effect in normal human lung fibroblasts. Apoptosis induced by EA was associated with decreased adenosine triphosphate (ATP) production; moreover, EA suppressed foetal bovine serum-dependent cell migration due to decreased levels of pro-matrix metalloproteinase-2 (pro-MMP-2 or gelatinase), pro-MMP-9 (or gelatinase B) and vascular endothelial growth factor-165 (VEGF-165) [29]. The Caco-2 cell line was also used to further characterize apoptosis induction by EA. The polyphenolic compound induced in these cells programmed cell death (via the intrinsic pathway), through Bcl-XL down-regulation, mitochondrial release of cytochrome c, activation of initiator caspase-9 and consequently of effector caspase-3 [30]. Similar results were obtained with HCT-15 and HCT-116 cell lines, where EA exposure promoted cell cycle arrest, induced apoptosis and increased the production of reactive oxygen intermediates [31,32].

Results from microarray analysis suggested that EA and urolithin-A and -B (Uro-A and Uro-B), at concentrations achievable in the gastrointestinal lumen from the diet, might contribute to colon cancer prevention by modulating gene expression. In fact, transcriptional profiling of Caco-2 cells exposed to EA and Uro revealed changes in the expression levels of genes involved in the mitogen-activated protein kinase (MAPK) signalling, such as growth factor receptors (fibroblast growth factor receptor-2, *FGFR2*; epidermal growth factor receptor, *EGFR*), oncogenes (*K-Ras*, *c-Myc*), tumour suppressors (*DUSP6*, *Fos*), and genes involved in cell cycle (*CCNB1*, *CCNB1IP1*) [33]. Microarray profiling of HCT-116 cells treated with EA also identified 857 differentially expressed genes, thus indicating that this compound might affect a large number of cell functions, such as proliferation, apoptosis, cell cycle and angiogenesis [32].

In vivo EA administration during 1,2-dimethylhydrazine (DMH)–induced colon carcinogenesis in rats resulted in a chemopreventive effect thanks to its anti-inflammatory properties, exerted by inhibition of NF-kB and subsequent iNOS, COX-2, TNF-α and IL-6 down-regulation [34]. In the same colon cancer model, EA induced apoptosis preventing PI3K/Akt signalling pathway activation and modulating downstream Bcl-2 family proteins. Bax expression and caspase-3 activation was also observed, leading to higher cytochrome c levels in the cytosol and eventually to cell death [35].

More recent studies have investigated the structure/antiproliferative activity relationship of EA derivatives against human colon cancer cells. The 4,4′-di-O-methylellagic acid (4,4′-DiOMEA) was the most effective agent in inhibiting colon cancer cell proliferation (13-fold more effective than other compounds of the same family); of interest, it was very active against colon cancer cells resistant to the chemotherapeutic agent 5-fluorouracil (5-FU) [36]. Also Uro-A, at concentrations achievable in the human colorectal tract, could sensitize colon cancer cells to 5-FU or its prodrug 5′-deoxy-5-fluorouridine (5′DFUR). Both 5-FU and 5′DFUR arrested the cell cycle at the S phase in the human colon cancer Caco-2, SW-480 and HT-29 cells and triggered apoptosis through the activation of caspase-8 and -9. Co-treatments with Uro-A decreased the half maximal inhibitory concentration (IC_50_) values for both 5-FU and 5′DFUR, by contributing to cell cycle arrest at the Gap 2/Mitosis checkpoint (G2/M) and increasing caspase activation. Thus, Uro-A would allow administration of lower 5-FU doses to achieve similar effects and this might in turn reduce systemic adverse effects [37]. These results suggest that structure-activity differences between EA and 4,4′-DiOMEA or Uro-A might constitute the basis for the development of more effective EA derivatives.

However, the clinical evidence of dietary polyphenols as CRC chemopreventive compounds is very weak. The first clinical trial in 35 CRC patients daily supplemented with pomegranate extracts has been recently conducted [38]. In particular, this study evaluated the expression of various CRC-related genes in normal and cancerous colon tissues before (biopsies) and after (surgical specimens) 5–35 days of pomegranate extract supplementation. The consumption of pomegranate extract was significantly associated with a counterbalance effect in the expression of genes modulated by the experimental protocol.

### 2.2. Ellagic Acid and Breast Cancer

Breast cancer is the most commonly occurring cancer in women and the second most common cancer overall [25,39]. About 5–10% of breast cancers are linked to inherited gene mutations (*BRCA-1* and *BRCA-2* mutations are the most common), while about 85% of cases occur in women who have no family history of breast cancer [40].

A first evidence of EA activity on breast cancer arises from a study assaying the effects on cell growth of an extract of *Terminalia chebula* fruit in several malignant cell lines, including human MCF-7 and mouse S115 cell lines. In all models, the extract decreased cell viability, inhibited cell proliferation, and induced cell death in a dose-dependent manner. Actually, EA was the phenolic compound present in the *T. chebula* fruit extract endowed with the highest growth-inhibitory effect [41].

The most common subtype of breast cancer is oestrogen receptor-positive, meaning that its growth can be hampered by drugs that block the production and/or the action of oestrogens. A study investigated whether EA might have estrogenic/anti-estrogenic activity and influence the activity of the oestrogen receptor subtypes ERα and ERβ. At low concentrations (0.3–30 ng/mL) EA displayed a mild but significant estrogenic activity via ERα, whereas it behaved as a full ERβ antagonist. Indeed, EA acted as a potent anti-estrogenic agent in the oestrogen-sensitive human breast cancer MCF-7 cells, increasing insulin-like growth factor-binding protein 3 (IGFBP-3) levels, whose production is usually inhibited by oestrogens [42]. Accordingly, previous finding demonstrated that EA in combination with 17β-oestradiol was able to counteract the hormone-induced increase in hTERT α+β + mRNA splice variant, while when used as single agent it increased hTERT α+β+ expression, confirming that EA possesses both estrogenic and anti-estrogenic effects in breast cancer cells [43]. These findings suggest that EA can be regarded as a natural selective oestrogen receptor modulator (SERM). Uro-A and Uro-B also showed estrogenic activity in a dose-dependent manner; therefore, similarly to EA, they act as potential endocrine-disrupting molecules that resemble other described “enterophytoestrogens” (microflora-derived metabolites with estrogenic/anti-estrogenic activity) [44].

TGF-β/Smads and PI3K signalling pathways are other well characterized mediators of EA activity against breast cancer. Co-treatment with EA and a specific inhibitor of Smad3 phosphorylation (SIS3) decreased the EA inhibitory activity on MCF-7 cell proliferation and reversed EA effects on the expression of TGF-β/Smad3 downstream targets [45]. The possibility that modulation of the TGF-β/Smads signalling pathway is involved in breast cancer cell cycle arrest induced by EA was reinforced by the results of gene expression profiling in the MCF-7 cell line. A cDNA microarray analysis identified a total of 4738 genes with a >2.0-fold change after 24 h treatment with EA, 16 of which belonged to the TGF-β/Smads signalling pathway [46]. Concerning the PI3K pathway, co-treatment of a panel of breast cancer cell lines with EA and the PI3K inhibitor GDC-0941 resulted in a significant decrease of cell growth, migration and invasion in vitro, as well as in reduced tumour initiation and metastasis formation in vivo [47].

Cancer stem cells are thought to be the origin of both primary and secondary breast tumours, and thus represent a critical target for both therapy and prevention. The murine breast cancer WA4 cell line contains a majority of cells possessing stem cell characteristics and has been used to examine the effects of a standardized extract of pomegranate. Proliferation of WA4 cells was inhibited by pomegranate extract in a time- and concentration-dependent manner, with induction of cell cycle arrest at the G0/G1 phase and apoptosis through caspase-3 activation. The major pomegranate extract components responsible for the observed inhibitory effects were EA, ursolic acid and luteolin [48]. A methanolic pomegranate peel extract also reduced cell proliferation and induced apoptosis in MCF-7 cells and, according to the results of HPLC analysis, the most abundant phenolic acid detected in the extract was EA [49].

It is well known that EA interferes with some angiogenesis-dependent pathologies. Wang and colleagues analysed its antiangiogenic effects in human breast cancer, revealing that in MDA-MB-231 xenografts EA significantly inhibited tumour growth and VEGF receptor 2 (VEGFR-2) phosphorylation [50]. A molecular docking simulation indicated that EA could form hydrogen bonds and aromatic interactions within the ATP-binding site of the VEGFR-2 kinase. These data support EA ability to counteract VEGFR-2 signalling pathway that accounts for its antiangiogenic potential [50]. Another study correlated the decrease in breast cancer cell migration mediated by pomegranate fruit juice (PFJ) or by a combination of three of its components (i.e., EA, luteolin and punicic acid) with modulation of gene expression. These compounds were found to: (a) inhibit the expression of genes involved in cell migration in response to stromal cells-derived factor 1-α (*SDF1-α*, a chemokine that attracts breast cancer cells to the bone), and (b) to stimulate the expression of genes that increase tumour cell adhesion [51].

Finally, a recent study has investigated EA effect on γ-irradiation of MCF-7 cells. Results suggested that tumour cell irradiation at the doses of 2- and 4-Gy, in the presence of EA micromolar concentrations, produced synergistic cytotoxic effects, as indicated by the increase of cell death and the decrease of colony forming ability. Combined treatment also resulted in a decrease of the mitochondrial membrane potential that favoured Bax upregulation and Bcl-2 downregulation, promoting apoptotic cell death [52]. Thus, EA appears as a potential adjuvant for improving the efficacy of cancer radiotherapy.

### 2.3. Ellagic Acid and Prostate Cancer

Prostate cancer is the fourth-most common cancer and the fifth leading cause of cancer death in men worldwide, with an incidence that differs by more than 25-fold among regions, the highest being Australia/New Zealand and the lowest South-Central Asia [25,53]. In Europe, prostate cancer is the second most common malignancy and the leading cause of cancer death in men. According to the American Cancer Society, 1 in 9 men are diagnosed with prostate cancer during their lifetime, and about 6 out of 10 cases are detected in men older than 65 [54].

In 2002, Narayanan and colleagues published the first study on EA and prostate cancer. Their genetic approach had the aim to identify alterations in gene expression in the human prostate cancer LNCaP cells after 48 h exposure to the polyphenolic compound. Their results revealed 593 genes with more than 2-fold differential expression, with respect to control. Alterations in p53-responsive genes, *p300*, *Apaf-1*, *NF-kB p50* and *p65* and *PPAR* families of genes were indicative of the multiple signalling pathways responsible for the observed growth inhibitory effects of EA in LNCaP cells [55].

Together with other components of pomegranate fruits (i.e., coffeic acid, lutheolin and punicic acid) EA has been shown to exert antiproliferative and caspase-dependent pro-apoptotic activities also against the human prostate cancer PC-3 cell line [41,56]. Recently, modulation of several signalling pathway proteins was found to be required for the EA effects in PC3 cells, such as decreased STAT-3, ERK and AKT phosphorylation in addition to increased levels of IL-6 [57].

Compared to its metabolite Uro-A, EA demonstrated a greater dose-dependent antiproliferative activity in PC-3 and DU-145 cells. EA induced apoptosis in both cell lines, while Uro-A exerted a pro-apoptotic effect only in DU-145 cells. A differential effect on the cell cycle progression was also observed: EA arrested cells in the S phase, while Uro-A induced G2/M arrest. Co-treatment with EA and Uro-A of PC-3 cells resulted in a more pronounced inhibition of cell proliferation [58]. Vanella and colleagues further investigated the molecular mechanisms of EA-induced apoptosis in LNCaP cells, revealing down-regulation of the antiapoptotic proteins silent information regulator-1 (SIRT-1), human antigen R (HuR) and heme oxigenase (ho-1). Moreover, EA treatment resulted in a significant increase of reactive oxygen species (ROS) levels and caspase-3 activation [59].

At non-toxic concentrations, EA acted as a potential inhibitor of the in vitro motility and invasive behaviour of PC-3 and rat prostate cancer PLS-10 cells [60,61]. This effect was attributed to a decreased secretion of MMP-2 and a reduced proteolytic activity of collagenase/gelatinase secreted by PLS-10 cells [61]. Confirming what previously demonstrated in breast cancer cells, the effects of PFJ components (EA, lutheolin, and punicic acid) included inhibition of prostate cancer cell chemotaxis toward SDF1-α. Also in the case of prostate cancer, a modulatory effect on gene expression, such as up-regulation of cell adhesion genes and down-regulation of genes promoting chemotaxis, has been considered involved in the migration inhibitory property of PFJ components [62]. A direct effect on angiogenic factors levels appears to be another mechanism through which EA exerts its antimetastatic potential against prostate cancer. In the LnCap model, EA reduced VEGF production and down-regulated the eicosanoid synthesis pathway as well as the ho-1 system, all involved in tumour growth and metastasis [63]. Wang and colleagues extended these in vitro observations to an in vivo immunodeficient murine model of prostate cancer, in which luciferase-expressing human PCa cells were injected subcutaneously near the prostate. By weekly monitoring tumour growth and spreading with bioluminescence imaging, the authors showed that EA, lutheolin and punicic acid combination efficiently inhibited metastasis formation and angiogenesis in vivo [64]. Another in vivo model (transgenic rat for prostate adenocarcinoma, TRAP) was used to investigate the chemopreventive effects of PFJ and EA on prostate carcinogenesis. After 10 weeks treatment, PFJ decreased the incidence of prostate adenocarcinoma and both EA and PFJ suppressed the progression of prostate carcinogenesis and induced caspase-3 dependent apoptosis [65].

Clinical trials have been carried out to evaluate EA as a supportive therapy for patients with prostate cancer. In particular, patients with hormone refractory prostate cancer were treated with a chemotherapy regimen containing estramustine phosphate and vinorelbine plus EA or with chemotherapy alone [66]. The results of this study indicated that EA reduced chemotherapy-associated myelotoxicity, in particular neutropenia, and induced a marked decrease in prostate-specific antigen (PSA) levels in a higher percentage of patients compared to chemotherapy alone. Moreover, patients receiving EA support therapy presented a positive trend in terms of objective response rate, although no significant differences in overall survival were observed between the two groups [66]. Another clinical trial assessed whether ETs from PFJ (i.e., EA and Uro) may reach the prostate after ingestion of ET-rich food. Sixty-three patients with prostate cancer were divided into three groups: control and consumers of walnuts or PFJ for three days before surgery. Independently of the ETs source, the main metabolites detected in the prostatic tissue were Uro-A-glucoronide and dimethyl-EA, which were considered the PFJ components responsible for EA beneficial effects against prostate cancer [67]. Moreover, a small phase II clinical study was performed in 69 patients to evaluate the influence of pomegranate extract on the levels of 8-hydroxy-2-deoxyguanosinelevel in the prostate tissue, as a marker of oxidative stress. Thirty-three and 36 men were randomized to receive orally pomegranate extract and placebo, respectively, for up to four weeks prior to radical prostatectomy. The results of this study confirmed the relative safety of short-term therapy with pomegranate extract; however, treatment did not result in a statistically significant protection from oxidative damage [68]. In another phase II study, pomegranate extract administration to 109 patients for 18 months was associated with ≥6 month increase in PSA doubling time without adverse effects [69]. Similar effects were previously observed when patients were treated with pomegranate juice [70].

Interestingly, prostate cancer represents the only case in which EA has been linked to differentiation of malignant cells, as demonstrated by the neuroendocrine differentiation of LnCap and DU145 cells. Differentiation of tumour cells into more mature normal-like cells represents a promising less toxic strategy, with respect to conventional chemotherapy. In fact, the results of this study demonstrated that EA possesses also pro-differentiation properties, which further reinforce its role as antitumor compound [71].

### 2.4. Ellagic Acid and Lung Cancer

Lung cancer is the most commonly diagnosed tumour worldwide (11.6% of the total cases) in both sexes [25]. It is the leading cause of cancer-related death (18.4% of the total) and has the lowest five-years survival rate (18%) [39]. The World Health Organization (WHO) classifies lung cancer into two subtypes: non-small cell lung cancer (NSCLC) and small cell lung cancer (SCLC). NSCLC represents 85% of cases of lung cancer and comprises adenocarcinoma, squamous-cell, and large-cell carcinoma, while SCLC represents 14–15% of all lung cancers [72]. Smoking accounts for more than 80% of all lung cancer cases, followed by pollutants, carcinogens, family history, radiation, age and diet.

The N-nitrosodiethylamine (NDEA)-induced lung cancer in mice was the first experimental model in which EA demonstrated antitumor activity. In the NDEA treated population it reduced tumour incidence from 72% to 20% and resulted to be more effective than quercetin, another dietary polyphenolic compound. The EA antioxidant properties contributed to the observed antitumorigenic effect, since in EA treated mice increased levels of reduced glutathione (GSH) and decreased lipid peroxidation were detected [73].

Recently, the anticancer effects of pomegranate leaf extract (PLE) were studied in vitro on the human NSCLC A549 and H1299 cell lines and mouse Lewis lung carcinoma LL/2 cell line. As observed in other cancer models, PLE inhibited cell proliferation in a time- and concentration-dependent manner, arresting cell cycle in the G2/M phase and promoting caspase-dependent apoptosis. In addition, PLE was able to counteract H1299 cells migratory and invasive behaviour, reducing MMP-2 and MMP-9 levels [74]. In the A549 cell line treated with pure EA, inhibition of the PI3K/Akt signalling pathway was identified as a possible molecular mechanism responsible for the antiproliferative potential of this compound [75].

Patients with NSCLC that harbour *EGFR* mutations (17.4% in Caucasians and 38.8% in Asians) may benefit from the treatment with EGFR tyrosine kinase inhibitors (TKI) [76]. However, primary or acquired resistance may limit the efficacy of therapies targeting mutated EGFR [77]. Different polyphenolic compounds, among which EA, have been tested for their ability to suppress TKI-resistance in a panel of HCC827 clones, including TKI-sensitive (TKIS) and -resistant (TKIR) cells. Jeong and colleagues showed that most of the polyphenols tested exerted strong antiproliferative effects against TKIR lung cancer cells, while they showed only modest activity against the TKIS ones. These results suggest that EA may not only inhibit lung cancer proliferation, but also overcome drug-resistance that frequently occurs during treatment with TKI [78].

### 2.5. Ellagic Acid and Melanoma

Malignant melanoma accounts for a low percentage of all skin cancer cases (<5%), but it has an extremely aggressive behaviour, which makes it the deadliest type of skin cancer [79]. Current therapeutic approaches against melanoma include surgical resection, chemotherapy, immunotherapy and targeted kinase inhibitors, applied as single agents or in combination, depending on the patient’s health, tumour location and stage. Although melanoma is curable if detected at an early localized form and the recently approved immunostimulating monoclonal antibodies have improved overall survival of patients with advanced disease, in most cases metastatic melanoma continues to have a dismal prognosis [80]. A specific biomarker of melanoma is tyrosinase, the rate-limiting enzyme required for the biosynthesis of melanin by melanocytes. EA was reported to react with copper, present in the active site of tyrosinase, altering its catalytic activity. Indeed, tyrosinase activity of B16 murine melanoma cells exposed to EA, recovered in a dose-dependent manner when copper ions were added to the medium. EA effects on melanogenesis were further investigated in vivo: brownish guinea pigs irradiated for two weeks with ultraviolet (UV)-light and locally treated with EA, showed suppression of skin pigmentation [81]. Further in vitro studies contributed to characterize EA mechanism of action against melanoma. In three human melanoma cell lines, 1205Lu, WM852c and A375, EA inhibited cell proliferation by promoting G1 cell cycle arrest, increased apoptosis, decreased IL-1β and IL-8 synthesis and NF-kB activity [82].

The EA antiproliferative potential against melanoma was also tested as polyphenolic whisky constituent: EA, gallic acid and lyoniresinol were the predominant whisky polyphenols and all of them significantly inhibited melanogenesis and tyrosinase activity in B16 murine melanoma cells [83]. Treatment with EA also inhibited extra-cellular matrix (ECM) invasion in response to VEGF-A by human melanoma M14 cells that express the VEGFR-2 [84]. In this study, VEGFR-2 negative and positive M-14 cell clones (M14-N and M14-NV, respectively) were used [85]. In fact, even though the invasiveness of M14-N and M14-NV cells is intrinsically high due to the expression of the VEGF-A co-receptor neuropilin-1 (NRP-1), EA inhibited VEGF-A induced ECM invasion only in the VEGFR-2 positive M14-NV cells [84,85,86,87].

Other studies have demonstrated the anti-melanogenic activity of EA derivatives, such as natural metabolites (Uro), artificial compounds resulting from chemical modifications of the EA molecule or innovative drug delivery systems carrying EA with no chemical modifications, with the aim to improve its solubility and bioavailability. Uro-A and Uro-B were found to suppress tyrosinase activity and to attenuate melanogenesis in B16 melanoma cells [88]. Moreover, an acetylated form of EA (EA-peracetate) inhibited murine melanoma B16 cell growth and induced apoptosis through Bcl-2 down-regulation. In vivo oral administration of EA-peracetate significantly suppressed melanoma growth in C57BL/6 mice and increased white blood cell number in peripheral blood. Both the antitumor efficacy and immune stimulation exerted by EA-peracetate in an in vivo model of melanoma were greater as compared to those induced by EA, suggesting that appropriate chemical modifications might enhance EA antitumor potential [89]. Finally, by using chitosan films as local carrier of EA, Kim and collaborators demonstrated that films containing 0.5 and 1% of the polyphenolic compound (*w*/*v*) exerted potent antiproliferative and apoptotic effects in human melanoma WM115 cells [90].

### 2.6. Ellagic Acid and Bladder Cancer 

Bladder cancer represents the ninth-most common cancer in the world, specifically the seventh most common in men, and the seventeenth most common in women [91]. The incidence of bladder cancer varies considerably according to the geographical area, with the highest incidence rates reported in Southern and Western Europe and North America [91].

The first study on EA antitumor potential against bladder cancer demonstrated the ability of the polyphenol to inhibit the enzymatic activity of N-acetyltransferase (NAT), an enzyme that acetylates aromatic amines involved in the aetiology of this tumour. Arylamine NAT activity and carcinogen (2-aminofluorene)-DNA adduct formation were investigated in vitro in the human bladder cancer T24 and TSGH8301 cell lines. In this model, EA inhibited both NAT activity and 2-aminofluorene-DNA adduct formation in a dose-dependent manner [92]. As demonstrated in other cancer types, EA reduced cell viability of T24 cells by induction of G0/G1-phase arrest and apoptosis. Moreover, EA increased p53 and p21 levels and decreased CDK2 expression, which may contribute to the observed G0/G1 arrest, and promoted caspase-3 activity [93]. Similar results were obtained in vitro with the human bladder cancer TSGH8301 cells [94].

Recently, we demonstrated that EA exerted antiproliferative activity in vitro against bladder cancer cells using a panel of four different human bladder cancer cell lines (i.e., T24, UMUC3, HT-1376 and 5637) and enhanced the cytotoxic effects of mitomycin C, a chemotherapeutic agent commonly used for the treatment of bladder cancer [84]. We also showed that EA inhibited VEGF-A–induced ECM invasion and chemotaxis, reducing VEGFR-2 expression, not only in bladder cancer cells, but also in human endothelial cells [95]. Moreover, EA down-regulated the expression of programmed cell death-ligand 1 (PD-L1), an immune checkpoint involved in tumour escape from immunological attack. Our study is, to date, the only one extending the in vitro results on EA anti-bladder cancer effects to an in vivo model. In particular, treatment with EA of nude mice bearing human bladder cancer xenografts significantly reduced tumour growth and spreading as well as tumour-associated angiogenesis [84].

Uro- A, Uro-B, 8-OMe-urolithin A, together with EA, were also tested in vitro against T24 cells. The results of this study showed that Uro-B IC_50_ values were similar to those of EA, and the authors suggested that EA and its metabolites induced caspase-3 mediated apoptosis and inhibited tumour cell proliferation by affecting the p38 MAPK and c-Jun pathways. The polyphenolic compounds reduced oxidative stress by decreasing the production of ROS and malondialdehyde and increasing superoxide dismutase (SOD) activity in H_2_O_2_-treated T24 cells [96]. Recently, the UMUC3 cell line was found highly susceptible to Uro-A, which promoted cell cycle arrest at the G2/M phase, increased apoptotic cell death and inhibited PI3K/Akt and MAPK signalling pathways [97]. Taken together, these studies emphasize the chemotherapeutic potential of Uro against bladder cancer and reinforce the idea that not only EA but also its metabolites could be beneficial for cancer treatment.

### 2.7. Ellagic Acid and Hepatocarcinoma 

Hepatocellular carcinoma (HCC) is the sixth most common cancer in the world, specifically the fifth most commonly occurring cancer in men and the ninth in women [98]. Generally, liver cancer is more common in the East Asia with, the highest incidence rate in Mongolia [99]. Its incidence is expected to increase until 2030 in some countries including the US, while in other countries, such as Japan, incidence has started to decline. Viral hepatitis B (HBV) and hepatitis C (HCV) are the most important causes of chronic liver disease and HCC [100].

An EA derivative, isolated from the acetone extract of a Chinese traditional herb from the family of Euphorbiaceae (3,3′-Di-*O*-methyl ellagic acid-4′-*O*-β-d-xylopyranoside, JNE2), was tested in vitro for its antitumor activity against the human HCC HepG2 cell line. This study provided the first evidence that an EA derivative was able to inhibit the proliferation of HCC cells in a dose- and time-dependent manner, block the cell cycle at the G1/S phase and hamper tumour invasiveness [101]. Moreover, EA was tested in HepG2 cells as radiosensitizer, by treating tumour cells with the polyphenol 12 h prior to γ-radiation. EA increased ROS generation and p53 expression and, at the same time, decreased the expression of several survival markers (i.e., p-AKT, p-NF-kB, p-STAT3). Combination of EA with radiation also blocked the cell population in the G2/M phase, induced apoptosis, and decreased the levels of IL-6, COX-2, TNF-α, MMP-9 [102].

Recently, to overcome the low solubility and bioavailability of EA, poly (lactic-co-glycolic) acid (PLGA) nanoparticles functionalized with chitosan and polyethylene glycol (PEG) were developed to encapsulate the polyphenolic compound, also with the aim of protecting them from the phagocytic uptake. All EA-containing nanoparticles exerted antiproliferative and apoptotic activity in HepG2 cells and, with the exception of EA-PLGA, the other formulations (i.e., EA-PLGA-CS and EA-PLGA-CS-PEG) reduced EA IC_50_ values of 1.5 and 2.7 folds, respectively [103].

The antioxidant and chemopreventive effects of EA was evaluated in vivo using the NDEA-induced hepatocarcinogenesis model in rats that is characterized by a significant decline in liver antioxidant enzymatic activities. Oral administration of EA at the dose of 50 mg/kg for 7 days before and 14 days after NDEA administration, produced a significant increase in antioxidant enzyme activity and serum total protein, concomitant with a decrease of tumour markers levels. EA administration for 21 days after NDEA administration, produced similar, although less pronounced, effects. Therefore, EA was able to scavenge free radicals, reduce liver injury and exert protective effects against oxidative stress, thus decreasing hepatocarcinogenesis [104]. Similar results were obtained by a recent study performed in the same experimental animal model. In fact, EA extracted from *Punica granatum* peels induced a significant decline in serum levels of hepatocarcinogenesis markers (i.e., α-fetoprotein, glypican-3, GPC-3, and STAT-3), a substantial increase in suppressors of cytokine signalling 3 (SOCS3) and a negative immunoreaction for VEGF in the liver tissue [105]. These in vivo studies confirmed EA pro-apoptotic, antiangiogenic and antiproliferative activities through which it may be beneficial in chemoprevention and treatment of HCC.

### 2.8. Ellagic Acid and Ovarian Cancer

Ovarian cancer encompasses a heterogeneous group of malignancies that vary in aetiology and molecular biology. Ninety percent of ovarian cancers are epithelial and the serous histological type has the highest lethality of all gynaecological cancers. Europe is the continent with the highest rates of ovarian cancer in the world with more than 42,700 deaths annually [106]. Chemotherapy for ovarian cancer usually is a combination of cisplatin and paclitaxel or docetaxel, typically involving 3 to 6 cycle of treatment, depending on the stage and type of the cancer.

Few studies, to date, have investigated EA effects on ovarian cancer. Treatment of the human ovarian carcinoma ES-2 and PA-1 cell lines with EA resulted in a dose- and time-dependent inhibition of cell proliferation, with G1 phase arrest of the cell cycle. Decreased levels of cyclins D1 and E and higher levels of p53 and p21 were reported to be responsible for the cell cycle arrest. EA also induced caspase-3 mediated apoptosis by decreasing the Bax/Bcl-2 ratio and inhibited autophagy. These effects contributed to increase human ovarian cancer cell sensitivity to doxorubicin [107].

The use of natural phenolic compounds like EA turned out to be a promising approach to prevent cisplatin resistance onset in ovarian cancer. A platinum-resistant subclone of the epithelial ovarian cancer A2780 cell line (A2780CisR) was obtained with 26 weekly cycles of treatment with cisplatin. Pre-treatment with EA of A2780CisR cells 48 h prior to cisplatin exposure moderately enhanced the cytotoxic effects induced by the platinum compound. Interestingly, treatment of A2780 cells with cisplatin in the presence of AE completely prevented the development of cisplatin resistance [108].

The A2780 cell line underwent inhibition of cell proliferation and migration upon exposure to PFJ, EA or lutheolin (another PFJ component) [109]. In addition, treatment with increasing EA concentrations resulted in a decrease of MMP-2 and MMP-9 expression. These results were confirmed in an in vivo experimental model (human ovarian cancer ES-2 cells heterotopically transplanted in nude mice) [109]. All treatments (i.e., PFJ, EA and lutheolin) inhibited tumour growth, with EA displaying a greater effect than lutheolin in decreasing MMP-2 and MMP-9 levels in vivo. These data confirm EA as a promising candidate for further preclinical studies on human ovarian cancer and as a potential adjuvant treatment for chemoprevention of ovarian cancer.

### 2.9. Ellagic Acid and Oral Cancer

Oral cancers develop on the tongue, tonsils and oropharynx, or gums, mouth floor, and other parts of the mouth. They represent the sixth most common cancers in the world [110]. In particular, in Europe, oral cancer is the 8th most frequent cancer and the 11th leading cause of cancer-related mortality, with estimated annual new cases close to 100,000 [39].

Evidence concerning EA activity against oral cancer derives from studies with black raspberry (BRB) components showing growth inhibitory effects on premalignant and malignant human oral cell lines. BRB components or differentially eluted fractions seemed to target signalling pathways regulating cell cycle progression [111]. A recent study further confirmed these data, demonstrating that EA induced apoptosis with caspase-3/7 activation and cleavage of poly-ADP ribose polymerase in human oral carcinoma HSC-2 cells [112].

To overcome EA poor water solubility and oral bio-availability EA-encapsulated chitosan nanoparticles were produced and tested as a potential drug delivery system in the human oral cancer KB cell line. This formulation exhibited significant cytotoxicity in a dose-dependent manner, with a very low IC_50_ value compared to the free EA IC_50_ (3.2-fold smaller), raising the possibility to overcome the EA pharmacokinetic limitations on oral cancer treatment [113].

The well characterized 7,12-dimethylbenz[a]anthracene (DMBA)-induced hamster buccal pouch (HBP) carcinogenesis in vivo model was adopted to investigate the effect of EA on the development of oral cancer. Dietary supplementation with 0.4% EA suppressed the HBP carcinoma formation by preventing the constitutive activation of Wnt pathway through downregulation of Fz, Dvl-2, GSK-3β and nuclear translocation of β-catenin, consequent to NF-kB inactivation. Modulation of key components of the mitochondrial apoptotic network was also observed [114]. The same hamster oral carcinogenesis model was used to investigate EA antiangiogenic effects. The polyphenolic compound significantly inhibited HIF-1α-induced VEGF/VEGFR-2 signalling by abrogating PI3K/Akt and MAPK activity via downregulation of PI3K, PDK-1, p-Akt (ser473), mTOR, p-ERK, and p-JNK [115]. Another carcinogen-induced rat oral cancer model was established to assay the ability of BRB and pure EA to inhibit oral lesion formation. F344 rats were treated with 4-nitroquinoline 1-oxide (4NQO) in drinking water for 14 weeks, to induce oral cancer formation. At week 14, 5–10% BRB or 0.4% EA were introduced in the rat diet. Histopathological analyses of the tumour mass demonstrated the ability of BRB and, to a lesser extent of EA, to inhibit tumour cell proliferation and progression, and to reduce mRNA levels of pro-inflammatory (Cxcl1, Mif, and Nfe2l2), antiapoptotic and cell cycle associated biomarkers (Birc5, Aurka, Ccna1, Ccna2) [116].

### 2.10. Ellagic Acid and Glioblastoma

Glioblastoma (GBM) is the most aggressive malignant primary brain tumour with an incidence 1.6 times higher in males compared to females. Globally, its incidence is highest in northern Europe [117]. In the US, GBM has an incidence rate of 3.19 per 100,000 persons. Survival from GBM is very poor; less than 5% of patients survive five years following diagnosis and survival rates have not substantially improved in the last three decades [118]. These data reinforce the idea that better strategies to prevent and treat GBM are strongly needed. The first two studies aimed at testing in vitro EA antiproliferative effects against GBM were performed using the human GBM U251, U87 and U118 cell lines [119,120]. The results showed a significant decrease of tumour cell proliferation and induction of apoptosis, due to S phase arrest and marked down-regulation of antiapoptotic proteins, respectively. Experiments performed in GBM xenografted mice confirmed the EA inhibitory effect on tumour growth that was associated with a marked decrease of Akt and Notch signalling pathways. EA also up-regulated the expression of E-cadherin and reduced the expression of Bcl-2, cyclin D1, cyclin-dependent kinase D2 (CDK2) and CDK6, Snail, MMP-2 and MMP-9. Overall, these data indicate that GBM treatment with EA can suppress brain tumour cells proliferation and invasive behaviour, encouraging further studies on this aggressive malignancy.

### 2.11. Ellagic Acid and Osteosarcoma

Osteosarcoma is not a common cancer: with about 500 new cases annually (half of which in children and teens), its incidence is less than 1% of total cancer cases, both in the US and in Europe [121]. Unfortunately, treatment of osteosarcoma has not essentially changed since the 1970s and outcomes are largely unimproved [122].

A first evidence of EA antitumor potential against osteosarcoma derived from an in vitro study, revealing a significant decrease of cell proliferation and increase of apoptosis induction, through chromosomal DNA fragmentation, Bax up-regulation and caspase-3 activation, in human osteogenic sarcoma cells [123]. Recently, there has been a renewed interest in EA as a potential drug candidate for the treatment of osteosarcoma. Exposure of the human osteosarcoma Saos-2 and MG63 cell lines to EA resulted in a marked inhibition of cell proliferation, with G1 phase arrest, as a consequence of reduced c-Jun expression. In addition, inhibition of cell migration and invasion was observed, suggesting that EA might exert antimetastatic effects [124]. These finding are particularly relevant, since metastasis remains the most important fatal complication of osteosarcoma.

## 3. Conclusions

For centuries, in ancient cultures, pomegranate fruit as well as its juice, extract, and oil have been used for the prevention and treatment of a multitude of diseases. To date, after decades of study, we know that EA, in addition to its antioxidant potential, exerts important antiinflammatory, antiproliferative, and antitumorigenic properties. The EA anticancer activity has been analysed both in in vitro and in in vivo in several types of cancers, which differ for clinical onset, genetic predisposition, risk factors and metastatic behaviour (Table 2 and Table 3). The results of a large number of studies indicate that EA, once entered into tumour cells, is able to arrest cell cycle progression, induce caspase-3 dependent intrinsic apoptotic pathway, modulate protein and gene expression and interfere with signalling pathways that regulate cell viability. Nevertheless, no cytotoxic effects have been detected when normal cells have been exposed to similar concentrations of EA.

At a systemic level, EA inhibits angiogenesis, cell migration and cell invasion, processes that are crucial for the infiltrative behaviour of tumour mass and the metastatic process. In addition, EA treatment increases tumour sensitivity to conventional therapy (i.e., chemotherapy and radiotherapy). Similar results have been obtained testing the anticancer activity of EA metabolites (Uro) and synthetic derivatives. Little information on the mechanism of action of these polyphenolic compounds is currently available. However, the hypothesized interference with the ATP binding to some receptor and/or non-receptor kinases might largely explain the observed EA effects on different signal transduction pathways.

To date, only few clinical studies have been conducted to evaluate the EA chemopreventive and therapeutic potential and most of them regard the introduction of pomegranate fruit and/or juice in cancer patient diet. Treatment with pomegranate extract or juice can be safely be administered in high doses. However, pomegranate components may interact with co-administered drugs due to inhibition of hepatic metabolism by cytochrome P450 [125]. Safety studies conducted in the rat model with UroA showed no modifications in clinical parameters, blood chemistry, haematology or no signs of organ toxicity. Moreover, in vitro and in vivo micronucleus assays demonstrated the lack of genotoxic effects after systemic exposure to UroA [126]. Of interest, novel drug-delivery systems that overcome the low EA solubility and bioavailability (i.e., biocompatible polymers-based nanoparticles/microcapsules/biofilms/micelles) were started to be tested in vitro and in vivo and are expected to fully develop the therapeutic potential of EA derivatives in the near future.

## Figures and Tables

**Table 1:** Foods and beverages containing ellagic acid.

Food or Beverage		Mean Content	S.D. ^a^	Reference
Berries	Black Raspberry, raw	38.00 mg/100 g FW ^b^	0.00	[14]
	Blackberry, raw	43.67 mg/100 g FW	24.54	[14]
	Cloudberry, raw	15.30 mg/100 g FW	0.00	[15]
	Red raspberry, raw	2.12 mg/100 g FW	8.35	[15]
	Strawberry, raw	1.24 mg/100 g FW	0.80	[16]
	Red raspberry, jam	1.14 mg/100 g FW	0.00	[17]
Berry juices	Black Muscadine grape, pure juice	0.90 mg/100 mL	0.95	[18]
	Green Muscadine grape, pure juice	0.93 mg/100 mL	0.78	[18]
	Red raspberry, pure juice	0.84 mg/100 mL	1.10	[19]
Tropical fruit juices	Pomegranate, pure juice	2.06 mg/100 mL	1.53	[20]
	Pomegranate, juice from concentrate	17.28 mg/100 mL	0.00	[20]
Nuts	Chestnut, raw	735.44 mg/100 g FW	240.69	[21]
	Japanese walnut	15.67 mg/100 g FW	7.64	[22]
	Walnut	28.50 mg/100 g FW	4.95	[22]
Alcoholic beverages	Walnut, liquor	1.22 mg/100 mL	0.42	[23]
	Cognac	1.13 mg/100 mL	1.42	[24]
	Rum	0.21 mg/100 mL	0.00	[24]
	Scotch, whisky	0.82 mg/100 mL	0.32	[24]
Mushrooms	*Fistulina hepatica*	2378 mg/kg DB ^c^	59,3	[2]

^a^ S.D., standard deviation; ^b^ FW, fresh weigh; ^c^ DB, dry basis.

**Table 2:** In vitro experimental evidence of ellagic acid (EA) therapeutic potential.

Formulation	Concentration	Biological Effect	Experimental Model	Reference
Pure EA	30 µg/mL; 7.5–37.5 µg/mL	Growth inhibition	Human colon cancer cell lines: SW480, HCT-116	[28,32]
	6–36 µg/mL	Cell cycle arrest in: G2/M phase	Human colon HCT-15 cancer cell line	[31]
Pomegranate leaf extract	6.25–200 µg/mL		Human NSCLC A549 and H1299 cell lines; mouse Lewis lung carcinoma LL/2.cell line	[74]
Pure EA	0.3–15 µg/mL; 1.5–22.5 µg/mL	G0/G1 phase	Human bladder cancer T24 and TSGH8304 cell lines	[93,94]
	3–30 µg/mL	G1 phase	Human ovarian carcinoma ES-2, PA-1 cell lines	[107]
	6 µg/mL		Human osteosarcoma Saos-2, MG63 cell lines	[124]
	15 µg/mL		Human melanoma 1205Lu, WM852c, A375 cell lines	[82]
	1.5–6 µg/mL		Human NSCLC A549 cell line	[75]
	15–30 µg/mL	G1/S phase	Human hepatocarcinoma HepG2 cell line	[101]
	4.5–18 µg/mL	S phase	Human glioblastoma U251, U87, U118 cell lines	[119]
			Human prostate cancer PC-3 and DU-145 cell lines	[56]
	0.3–9 µg/mL; 6–36 µg/mL; 7.5–37.5 µg/mL	Apoptosis induction, via intrinsic apoptotic pathway activation	Human colon cancer cell lines: Caco-2; HCT-15; HCT-116	[30,31,32]
	3–30 µg/mL		Human ovarian carcinoma ES-2, PA-1 cell lines	[107]
	15–30 µg/mL		Human glioblastoma U251, U87, U118 cell lines	[119]
	7.5–30 µg/mL		Human melanoma 1205Lu, WM852c, A375 cell lines	[82]
	15–45 µg/mL		Human oral carcinoma HSC-2 cell line	[112]
	0.3–15 µg/mL; 1.5–22.5 µg/mL		Human bladder cancer T24 and TSGH8304 cell lines	[93,94]
	3–30 µg/mL; 0–30 µg/mL; 4.5–18 µg/mL		Human prostate cancer PC-3, LNCaP, DU-145 cell lines	[56,57,58,59]
Pomegranate leaf extract	6.25–200 µg/mL		Human NSCLC A549 and H1299 cell lines; mouse Lewis lung carcinoma LL/2 cell line	[74]
Pure EA	30 µg/mL	Inhibition of tumour cell migration, invasion, metastasis, due to MMP-2, MMP-9, VEFGs downregulation	Human colon cancer Caco-2 cell line	[30]
	6 µg/mL		Human osteosarcoma Saos-2 and MG63 cell lines	[124]
Pomegranate juice; EA; Lutheolin	5–10%; 5–15 µg/mL; 5–15 µg/mL		Human ovarian cancer A2780 cell line	[109]
	3–9 µg/mL		Human bladder cancer cell lines: T24, UMUC3,5637, HT-1376	[84]
Pure EA	4 µg/mL; 7.5–30 µg/mL		Human PC-3 and rat PLS-10 prostate cancer lines	[60,61]
	6.25–200 µg/mL		Human NSCLC H1299 cell line	[74]
	3 µg/mL	Enhanced sensibility to radiotherapy	Human breast cancer MCF7 cell line	[52]
	3 µg/mL		Human hepatocarcinoma HepG2 cell line	[102]
Pomegranate juice; Lutheolin+EA+ punicic acid	1–5%; 1–8 µg/mL; 4–8 µg/mL	Inhibition of genes promoting tumour cell migration and up-regulation of genes promoting cell adhesion	Human breast cancer MDA-MB-231 and MCF7 cell lines	[51]
Pure EA	7.5–15 µg/mL		Human prostate cancer DU145, PC3, LNCaP cell lines	[62,63]
	30 µg/mL	DNA damage, p53 and p21 activation, IGF-II downregulation; Increased production of ROS	Human colon cancer SW480 and HCT-115 cell lines	[28,31]
	3–30 µg/mL		Human ovarian carcinoma ES-2 and PA-1 cell lines	[107]
	0.3–15 µg/mL		Human bladder cancer T24 and TSGH8301 cell lines	[82,83]
	30–36 µg/mL	Neuroendocrine differentiation of cancer cells	Human prostate cancer LnCap and DU145 cell lines	[57]
	3–30 µg/mL	Overcome of TKI-resistance	Human NSCLC HCC827 clones	[76]
	0.96–3 µg/mL	Enhanced sensibility to doxorubicin and cisplatin	Human ovarian cancer ES-2, PA-1, A2780 cell lines	[107,108]
	1.5–18 µg/mL	Enhanced sensibility to mitomycin C	Human bladder cancer T24, UMUC3, 5637, HT-1376 cell lines	[84]
	0.3–30 ng/ml; 0.3 µg/mL–3 ng/mL	Estrogenic; anti-estrogenic activity	Human breast cancer MCF-7 cell line	[42,43]
	3–9 µg/mL; 15 µg/mL	Modulation of TGF-β; Smads and PI3K pathways	Human breast cancer MCF-7 cell line	[45,46,47]
	6–18 µg/mL	Down-regulation of PDL-1	Human bladder cancer T24, UMUC3,5637, HT-1376 cell lines	[84]
	30 µg/mL	Changes in gene expression	Human colon cancer HCT-116 and Caco-2 cell lines	[32,33]
EA; metabolites UroA and UroB	3 µg/mL; 12 µg/mL		Human prostate cancer LNCaP cell line	[58]
EA, UroA, UroB, 8-OMe-urolithin A	30 µg/mL	Antiproliferative effect by p38-MAPK and/or c-Jun medicated caspase-3 activation	Human bladder cancer T24 and UMUC3 cell lines	[96,97]
EA and UroA, UroB, UroC	0.3–36 µg/mL	Cell cycle arrest, increasing apoptotic cell death and inhibiting PI3K/Akt and MAPK signalling pathway	Human NSCLC A549 cell line	[75]
UroA and UroB	0.15–22.5 µg/mL	Inhibition of viability and tysosinase activity	Murine melanoma B16 cell line	[88]
UroA	0.075–60 µg/mL	Estrogenic activity	Human breast cancer MCF-7 cell line	[44]
EA derivative 4,4′-DiOMEA	3 µg/mL	Inhibition of cell proliferation, enhancing sensibility to 5-FU and 5’DFUR	Human colon cancer SW-620, SW-620-5FuResistant, HT-29, Caco-2, SW-480 cell lines	[36,37]
EA-encapsulated chitosan nanoparticles	0.03–15 µg/mL	Antiproliferative/apoptotic effects	Human oral cancer KB cell line	[113]
	3–30 µg/mL		Human hepatocarcinoma HepG2 cell line	[103]
Chitosan-EA films	0.3–15 µg/mL		Human melanoma WM115 cell line	[90]
EA-peracetate	0.2–100 µg/mL		Murine melanoma B16 cell line	[89]
Pomegranate extract	0.5–1% (*w*/*v*); 5–200 µg/mL		Murine breast cancer WA4 cell line	[48]
			Human breast cancer MCF-7 cell line	[49]
*T. chebula* fruit extract	EA IC_50_ = 23.5 µg/mL		Human MCF-7 and mouse S115 breast cancer cell lines, human osteosarcoma HOS-1 cell line, human prostate cancer PC-3 cell line	[41]

**Table 3:** In vivo preclinical and clinical evidence of ellagic acid (EA) therapeutic potential.

	Formulation	Dose	Biological Effect	Experimental Model	Reference
Preclinical studies	Pure EA	60 mg/kg body weight	Chemopreventive effect	DHM-induced colon carcinogenesis in rats	[34]
	Pomegranate fruit juice (PFJ)	60 mg/kg body weight		Transgenic rat for adenocarcinoma of prostate (TRAP) model	[65]
		50 mg/kg body weight		NDEA-induced hepatocarcinogenesis in rats; NDEA induced lung carcinogenesis in mice	[73,104,105]
		12 µg/mL		DHM-induced colon carcinogenesis in rats	[35]
		60 mg/kg body weight	Apoptosis induction through AKT-PI3K pathway inhibition	DMBA-induced hamster buccal pouch carcinogenesis model	[115]
				Glioblastoma xenografted mice	[120]
		0.1–0.4% of the diet	Suppressed carcinogenesis through Wnt pathway inactivation	Human breast cancer MDA-MB-231 xenograft	[51]
	Pure EA	40 µg/g body weight	Inhibition of tumour growth and angiogenesis	Bladder cancer xenografts in nude mice	[84]
		50 or 100 mg/kg		Immunodeficient murine model of prostate cancer	[64]
	EA+lutheolin+punicic acid from pomegranate extract	40 mg/kg		DMBA-induced hamster buccal pouch carcinogenesis model	[115]
	EA; Lutheolin; pomegranate fruit juice	64 µg/component/day; 50 mg/kg body weight; 20 ml/kg body weight		Nude mice heterotopically transplanted with human ovarian ES-2 cancer cells	[109]
	EA dispersion (1% *w*/*v*) in water; propyleneglycol (10:90 *v*/*v*)	20 µL, topically	Suppression of melanogenesis	Brownish-guinea pigs UV light-irradiated; B16 melanoma cells-inoculated C57BL/l mice	[81]
	EA and black raspberries	0.4%; 5–10% of the diet	Inhibition of tumour cell proliferation and reduced levels of pro-inflammatory biomarkers	4NQO-induced rat oral cancer model	[116]
Clinical studies	Pomegranate extract	900 mg	Modification in gene expression pattern	Colorectal patients	[38]
	Pomegranate extract	2000 mg daily	No change in oxidative damage	Prostate cancer patients	[68]
	Pomegranate extract	1000 or 3000 daily	Prolongation of PSA doubling time	Prostate cancer patients	[69]
	Pure EA		Better response to traditional drugs and reduced systemic toxicity	Prostate cancer patients	[66]
	Pomegranate juice	180 mg daily	Protective effect against cancer	Prostate cancer patients	[67]
	Pomegranate juice	90–450 mL daily	Prolongation of PSA doubling time	Prostate cancer patients	[70]
